# GINOM: A statistical framework for assessing interval overlap of multiple genomic features

**DOI:** 10.1371/journal.pcbi.1005586

**Published:** 2017-06-15

**Authors:** Darshan Bryner, Stephen Criscione, Andrew Leith, Quyen Huynh, Fred Huffer, Nicola Neretti

**Affiliations:** 1 Naval Surface Warfare Center Panama City Division, Panama City, Florida, United States of America; 2 Department of Molecular Biology, Cell Biology and Biochemistry, Brown University, Providence, Rhode Island, United States of America; 3 Center for Computational Molecular Biology, Brown University, Providence, Rhode Island, United States of America; 4 Institute for Brain and Neural Systems, Brown University, Providence, Rhode Island, United States of America; 5 Department of Statistics, Florida State University, Tallahassee, Florida, United States of America; University of Tokyo, JAPAN

## Abstract

A common problem in genomics is to test for associations between two or more genomic features, typically represented as intervals interspersed across the genome. Existing methodologies can test for significant pairwise associations between two genomic intervals; however, they cannot test for associations involving multiple sets of intervals. This limits our ability to uncover more complex, yet biologically important associations between multiple sets of genomic features. We introduce GINOM (Genomic INterval Overlap Model), a new method that enables testing of significant associations between multiple genomic features. We demonstrate GINOM’s ability to identify higher-order associations with both simulated and real data. In particular, we used GINOM to explore L1 retrotransposable element insertion bias in lung cancer and found a significant pairwise association between L1 insertions and heterochromatic marks. Unlike other methods, GINOM also detected an association between L1 insertions and gene bodies marked by a facultative heterochromatic mark, which could explain the observed bias for L1 insertions towards cancer-associated genes.

## Author summary

The age of genomics has made a large number of datasets available for the wider scientific community. Many of these datasets come in the form of genomics tracks, represented as features associated with a collections of genomic intervals along chromosomes. A common talk in genomics is to identify putative associations between these features that can lead to new insights about genome organization and function. For example, activity of certain classes of genes might be influenced by the presence of specific combinations of chromatin modifications and binding of transcription factors at their promoters or enhancers. Here, we present a novel methodology, named GINOM (Genomic INterval Overlap Model), to test for the significance of these associations. We apply it to the problem of detecting biases of the locations along chromosomes where mobile genetic elements tend to insert themselves, and identify a potential preference for L1 elements towards gene bodies marked by a facultative heterochromatic mark.

## Introduction

A fundamental question in genome biology is whether two or more genomic features, for example gene promoters/bodies and histone modifications, are associated. These associations can shed light on fundamental regulatory mechanisms, such as the epigenetic regulation of gene expression. Genomic features can be represented as intervals, and thus the question becomes whether one set of genomic intervals, the query set, overlaps another set or sets of intervals, the reference set(s), significantly more or less than what would be expected by chance. The results of this test can guide the exploration of the underlying nature of the association; for example, if a histone modification is found to be associated with promoters of transcribed genes, one might hypothesize that the modification is required for active transcription. Thus, there is a widespread need for an accurate and computationally-efficient statistical test of genomic interval overlap.

Several statistical strategies to examine genomic interval overlap have been developed [1–5]. One study tested for associations of transposable element insertions in the genome with various epigenomic features [1]. Using transposon mutagenesis, [1] created a database of transposable element insertions across the mouse genome and subsequently tested for insertion site bias with respect to a randomized control set. Another method, MULTOVL, performs a Monte Carlo shuffling of the intervals uniformly throughout the genome to obtain an empirical null distribution of overlap lengths in order to test for significance [2]. The Binary Interval Search (BITS) algorithm also uses a Monte Carlo simulation by uniformly shuffling the query intervals many times and obtaining an empirical null distribution of the intersection count [3]. The Genome Association Test (GAT) presented in [4] is another statistical test of overlap length based on Monte Carlo simulations as in [2], but the randomization procedure used to form this empirical null distribution can be designed to exclude regions such as gaps and repetitive sequences. Another method called GenometriCorr is an R package that includes four different statistical tests for spatial relationships of genomic intervals [5]. Two of the tests (the absolute distance test and the Jaccard test) rely on Monte Carlo randomization to formulate and test against an empirical null, and the other two tests (the relative distance test and the projection test) use analytical null distributions. The main limitation of these statistical strategies is their pairwise treatment of genomic intervals, where a query interval set is compared to one or multiple reference sets individually. These methods cannot reveal any higher order associations, i.e. any association between a query interval set and multiple reference sets simultaneously.

We present a robust statistical framework called GINOM (Genomic INterval Overlap Model) that adds more flexibility to the study of associations between genomic intervals. We impose a parameterized probability model, i.e. a density function, on query interval location with respect to any number of reference sets. Given query interval data, the model parameters are estimated through likelihood-based methods. Each parameter value is interpreted as the amount of departure from the null distribution on the genomic loci and is indicative of the query interval overlap with a certain reference set or group of reference sets. To specifically address the inclusion of higher order associations, we design the model in GINOM to consider any possible combination of reference sets rather than restricting to only pairwise comparisons. Since it is possible that some combinations will have no effect on query interval location, we provide an automatic selection procedure to keep only the model terms that best describe the data.

## Methods

Here we define a statistical model, i.e. a probability density, of the location of a query interval with respect to multiple sets of reference intervals. We design the model to reflect the tendencies of a query interval to overlap a reference set or a combination of reference sets more or less than would be expected according to a predefined null distribution. In this section we explicitly formulate the model equation and explain how to estimate and interpret the parameter values given a set of query intervals. Furthermore, we discuss hypothesis testing and restricting the number of model parameters through model selection.

### Notation and problem setup

Our goal is to define the probability density function of a query interval starting point location conditional on a given query interval length with respect to known sets of reference intervals. This density will be defined over all possible genomic loci and formulated as a mixture of deviations from a given null distribution. These deviations from the null occur only on the genomic index sets that would indicate an overlap of a query interval of the given length with one or more of the reference sets.

Define the discrete set of nucleotides that comprise an organism’s genome as G={1,…,L}, where *L* is the length of the genome. A query interval q(X,Y)⊆G is defined from two discrete random variables—the starting point location *X* and the length (or cardinality) *Y*—and is given as *q*(*X*, *Y*) = {*X*, *X* + 1, …, *X* + *Y* − 1}. From here onward, we use the notation of capital *X* and *Y* when referring to the random variables and lower case *x* and *y* when referring to observations of the respective random variables. Since the distribution of query interval starting points depends on the query interval length, we are concerned with modeling the conditional distribution of *X*|*Y*. We denote *f*(*x*|*y*) as the probability density function of *X*|*Y*.

Let *R*_1_, *R*_2_, …, *R*_*N*_ be *N* known reference sets, where each *R*_*i*_ is defined as the union of a set of intervals on G. In practice, each *R*_*i*_ is set to represent a particular feature of the genome that may influence the distribution of *X*|*Y*; for example, *R*_*i*_ could consist of all the loci that lie within genic regions. We say that a query interval *q*(*x*, *y*) overlaps *R*_*i*_ if the intersection of *q*(*x*, *y*) and *R*_*i*_ is non-empty. Let *r*_*i*_(*x*|*y*) be the overlap indicator function for *q*(*x*, *y*) and *R*_*i*_, which is given by

$$\begin{array}{c} r_{i}\left(x|y\right)=\left\{\begin{array}{cc} 1, & \text{if}\;\;q\left(x,y\right)\cap R_{i}\neq \emptyset \\ 0, & \text{otherwise}. \end{array}\right. \end{array}$$ (1)

In other words, *r*_*i*_(*x*|*y*) equals 1 over all *x* such that a query interval *q*(*x*, *y*) would overlap *R*_*i*_, and it equals 0 over all *x* where *q*(*x*, *y*) would not overlap *R*_*i*_.

Note that it is possible for a query interval to overlap more than one reference set at a time; therefore, we must define the overlap indicator function for multiple reference sets. Consider a non-empty subset of reference set indices given by *π* ⊆ {1, 2, …, *N*}. For example, if *N* = 3, all possible values of *π* are {1}, {2}, {3}, {1, 2}, {1, 3}, {2, 3}, and {1, 2, 3}. The overlap indicator function indexed by the set *π* is defined as *r*_*π*_(*x*|*y*) = ∏_*i*∈*π*_ *r*_*i*_(*x*|*y*). It is the indicator function of the simultaneous overlap of *q*(*x*, *y*) with all the reference sets given in the index set *π*. E.g. if *π* = {1, 2, 3}, then *r*_{1,2,3}_(*x*|*y*) equals 1 over all values of *x* where *q*(*x*, *y*) would overlap reference sets *R*_1_, *R*_2_, and *R*_3_ simultaneously and equals 0 otherwise.

Suppose further that, conditional on the length *Y* = *y*, we have defined a null distribution *f*_0_(*x*|*y*) of query interval starting point locations. For example, the null distribution could be defined as the discrete uniform distribution over $G_{0}\left(y\right)\subseteq G$, the set of all *x* where *q*(*x*, *y*) would lie completely within the mappable regions of G. That is,

$$\begin{array}{c} f_{0}\left(x|y\right)=\left\{\begin{array}{cc} 1/|G_{0}\left(y\right)| & \text{if}\;x\in G_{0}\left(y\right)\\ 0 & \text{otherwise}, \end{array}\right. \end{array}$$ (2)

where | ⋅ | denotes set cardinality. This null distribution suggests that a query interval has an equal chance of lying anywhere inside the mappable regions of the genome and no chance of lying outside. The restriction to the mappable regions is due to the impossibility of observing a query interval in any non-mappable region. Although we develop the theory behind GINOM to incorporate any arbitrary null distribution, in practice we use the null provided above in Eq (2).

### Model formulation and interpretation

In our model we take the density of a query interval to be

$$\begin{array}{c} f\left(x|y\right)=c\left(\theta ,y\right)f_{0}\left(x|y\right)\exp\left\{\sum_{\pi }\theta_{\pi }r_{\pi }\left(x|y\right)\right\}, \end{array}$$ (3)

where *c*(*θ*, *y*) is the normalizing constant and the summation is over the non-empty subsets of {1, 2, …, *N*}, i.e. all possible combinations of reference sets. Here, *θ* is the vector of model parameters. We choose a model density with log-linear form for various reasons. First, since the equation is formulated as an additive mixture of effects (in the logarithmic sense), model interpretation is intuitive and achieved through examining the value of *θ*. The components of *θ* describe different types of departures from the null distribution. As *θ*_*π*_ increases (decreases), fixing all other components of *θ*, the probability that a random query interval intersects all of the reference sets in *π* increases (decreases). As *θ*_*π*_ approaches infinity (negative infinity), fixing all other components of *θ*, the probability approaches 1 (0). A further advantage to the log-linear form is that the model equation belongs to an exponential family of distributions. This distribution family satisfies many regularity conditions that allow for a straightforward application of various techniques for statistical inference and hypothesis testing. Finally, in this formulation each component of *θ* can take any value in R, and thus an optimization over *θ* is unconstrained and computationally more efficient than a constrained optimization.

One can use *θ* to define a query interval enrichment profile across the genome in the following manner. The ratio $f\left(x|y\right)/f\left(\tilde{x}|y\right)$ gives the relative likelihood of a random query interval of length *y* having its left endpoint at *x* versus another location $\tilde{x}$. Comparing the model in Eq (3) with the null distribution *f*_0_, we say that the endpoint *x* has been *enriched* (*depleted*) relative to $\tilde{x}$ under our model if the ratio $f\left(x|y\right)/f\left(\tilde{x}|y\right)$ is greater than (less than) $f_{0}\left(x|y\right)/f_{0}\left(\tilde{x}|y\right)$. The function

$$\begin{array}{c} h\left(x|y\right)=\sum_{\pi }\theta_{\pi }r_{\pi }\left(x|y\right), \end{array}$$ (4)

which is a summation of certain components of *θ*, may be regarded as giving an enrichment profile. If *h*(*x*|*y*) is greater than (less than) $h(\tilde{x}|y)$, then it follows that $f\left(x|y\right)/f\left(\tilde{x}|y\right)$ is greater than (less than) $f_{0}\left(x|y\right)/f_{0}\left(\tilde{x}|y\right)$, and thus *x* is enriched (depleted) relative to $\tilde{x}$. Moreover, the ratio between the two ratios above, $\left(f\left(x|y\right)/f\left(\tilde{x}|y\right)\right)/\left(f_{0}\left(x|y\right)/f_{0}\left(\tilde{x}|y\right)\right)$, is equal to $\exp\{h\left(x|y\right)-h\left(\tilde{x}|y\right)\}$. This quantity provides a measure of the degree of enrichment (depletion) at *x* relative to $\tilde{x}$ under our model. Using this expression, it may be seen that increasing (decreasing) the value of *θ*_*π*_ increases (decreases) the degree of enrichment for all locations *x* where *q*(*x*, *y*) intersects all of the reference sets in *π* relative to all other locations $\tilde{x}$.

Note that the expressions presented in the above paragraph simplify when using the standard null distribution provided in Eq (2). Assuming that locations *x* and $\tilde{x}$ are both mappable, then $f_{0}\left(x|y\right)/f_{0}\left(\tilde{x}|y\right)=1$. We say that *x* is enriched (depleted) with respect to $\tilde{x}$ if $f\left(x|y\right)/f\left(\tilde{x}|y\right)=\exp\left\{h\left(x|y\right)-h\left(\tilde{x}|y\right)\right\}$ is greater than (less than) 1, i.e. if *h*(*x*|*y*) is greater than (less than) $h(\tilde{x}|y)$. Typically, we are interested in studying the enrichment of *x* with respect to a mappable $\tilde{x}$ such that $q(\tilde{x},y)$ would not overlap any of the reference sets. In this case of $\tilde{x}$ in the so-called “background” portion of the genome, $h(\tilde{x}|y)=0$, and, therefore, *x* is enriched (depleted) simply if *h*(*x*|*y*) is positive (negative). From this point onward, we exclusively use this simplified condition when determining the enrichment of *x*.

To help with the above model interpretation, we provide a simple example with *N* = 2 and *f*_0_ as in Eq (2). Since *N* = 2, the model terms are given by the set indices *π* = {1}, {2}, {1, 2}. Suppose that *θ* = (*θ*_{1}_, *θ*_{2}_, *θ*_{1,2}_)′ = (0.5, 0.7, − 0.2)′, where the prime denotes vector transpose. Now, for an *x* such that *q*(*x*, *y*) overlaps *R*_1_ but not *R*_2_, the enrichment is given by *h*(*x*|*y*) = *θ*_{1}_ = 0.5, which is positive. Thus, the set of all such *x*’s are enriched, and the probability of *q*(*x*, *y*) is *e*^0.5^ = 1.65 times greater than $q(\tilde{x},y)$, where $\tilde{x}$ is in the background. Similarly, the set of all *x*’s such that *q*(*x*, *y*) overlaps *R*_2_ but not *R*_1_ are enriched, and the probability of *q*(*x*, *y*) is *e*^0.7^ = 2.01 times that of $q(\tilde{x},y)$. For an *x* such that *q*(*x*, *y*) overlaps both *R*_1_ and *R*_2_, the enrichment is given by *h*(*x*|*y*) = *θ*_{1}_ + *θ*_{2}_ + *θ*_{1,2}_ = 1.0. In this case, the probability of *q*(*x*, *y*) is *e*^1.0^ = 2.72 times that of $q(\tilde{x},y)$. Notice that even though the individual model parameter *θ*_{1,2}_ is less than zero, there is still enrichment in these *x*’s rather than depletion. The model parameter *θ*_{1,2}_ represents the interaction effect of *R*_1_ and *R*_2_—the effect of *R*_1_ and *R*_2_ beyond that of simply adding the two individual effects together. In this case, the overall effect of *R*_1_ and *R*_2_ is slightly less than their additive effect due to the negative interaction term. For more details on model interpretation, see the Model Interpretation section of S1 Text.

In general, since there are 2^*N*^ − 1 possible non-empty subsets of {1, 2, …, *N*}, there are that many possible model parameters in Eq (3). In practical implementations, 2^*N*^ − 1 can be quite a large number, and the inclusion of all possible parameters can yield an unnecessarily complex model. Therefore, to avoid overfitting, we typically restrict to a smaller submodel by setting the less important parameters equal to zero and thus effectively dropping those terms from the model equation. For example, for ease of interpretation, we could decide that any term with third-order interactions or higher—that is, if *π* contains three or more reference set indices—is too complex to include in the model. Thus, we would automatically exclude those terms from consideration in the model equation, and the summation would be over all *π* such that |*π*| ≤ 2. From here on, we denote *d* as the number of model parameters included in the model. If all possible parameters are included in the model, i.e. if *d* = 2^*N*^ − 1, then we say that *f* is the full model.

### Parameter estimation, hypothesis testing, and model selection

Suppose we are given data in the form of *n* query intervals {*q*(*x*_*i*_, *y*_*i*_), *i* = 1, …, *n*}, and let us assume that the *x*_*i*_’s are conditionally independent given the *y*_*i*_’s. In order to fit the model to the data, we seek the maximum likelihood estimate (MLE) of *θ* given the data. To compute the MLE $\hat{\theta }$, we maximize over the likelihood function, which is equal to the joint density function

$$\begin{array}{c} \prod_{i=1}^{n}f\left(x_{i}|y_{i}\right)=\left(\prod_{i=1}^{n}c\left(\theta ,y_{i}\right)f_{0}\left(x_{i}|y_{i}\right)\right)\exp\left\{\theta^{\prime }T\right\}, \end{array}$$ (5)

where the prime denotes vector transpose and *T* is a length *d* vector with the *π*-th component given by $t_{\pi }=\sum_{i=1}^{n}r_{\pi }\left(x_{i}|y_{i}\right)$. The value *t*_*π*_ is the number of query intervals in the dataset that overlap all of the reference sets given in the index set *π* simultaneously. After taking the logarithm of the above function and dropping terms that are constant in *θ*, we are left with the following objective function:

$$\begin{array}{c} \ell \left(\theta \right)=\theta^{\prime }T+\sum_{i=1}^{n}\log\left(c\left(\theta ,y_{i}\right)\right). \end{array}$$ (6)

The MLE is thus computed as

$$\begin{array}{c} \hat{\theta }=\underset{\theta \in R^{d}}{\text{argmax}}\left\{\ell (\theta )\right\}. \end{array}$$ (7)

See S1 Text for an efficient method to compute the optimization in Eq (7) as well as approximate confidence intervals for each component of *θ*.

Now that we have a means for computing the MLE, and since the density *f* satisfies all the necessary regularity conditions, we can use the generalized likelihood ratio test (GLRT) for hypothesis testing (see Ch. 8 of [6]). In particular, we use the GLRT to test whether a specific component of *θ* differs from zero or not, i.e. whether a certain reference set or combination of reference sets affects query interval location. In order to perform this statistical test for the *π*-th component, one must solve Eq (7) twice—once allowing *θ*_*π*_ to be unconstrained in the optimization and once with the constraint that *θ*_*π*_ = 0. If the value of $\ell (\hat{\theta })$ changes enough with addition of this constraint, i.e. with the removal of the *π*-th model term, then we claim that *θ*_*π*_ is not equal to zero. For more detailed information on the GLRT, including the computation of a *p*-value for the test, see S1 Text.

In order to select the model parameter configuration that best describes the data, we implement the widely-used bidirectional stepwise model selection algorithm [7]. This algorithm systematically adds and removes model terms one at a time in an effort to find a configuration that minimizes the Bayesian Information Criterion (BIC) [8], which is given by $BIC=d\log n-2\ell (\hat{\theta })$. The BIC is a log-likelihood function that is penalized by the number of model parameters; therefore, the model configuration with optimal BIC is one that offers a high likelihood value with a small number of model parameters. The result of the stepwise algorithm is a model that contains only the most influential parameters, allowing for an easy yet biologically meaningful model interpretation.

## Results/Discussion

In what follows, we first apply GINOM to *in silico* data to demonstrate its performance and compare its predictive ability to that of currently available software. We then apply it to a real biological dataset and again compare its performance to the same software.

### Simulated query intervals

We analyzed the performance of GINOM on query interval sets simulated directly from the model equation Eq (3) using the nine reference sets listed in Table 1. This is the same set of features used in the LINE-1 insertion bias study we discuss later and contains a combination of genomic and epigenomic features associated with genome accessibility and transcriptional regulation. For simplicity in our simulations, we fix the query interval length to be *Y* = 1 unless stated otherwise and assume all loci in G are mappable when forming the null distribution in Eq (2). A simulation is done by first selecting *N* reference sets to consider, then optionally restricting the domain to a subset of the entire genome, followed by setting the true values for each component of *θ*, and then finally generating a random sample of size *n* via acceptance/rejection algorithm. The result of the simulation is a set of *n* query intervals that are independently and identically distributed according to the specified model.

**Table 1:** List of reference sets.

Name	Index
H3K4me3	1
gene bodies	2
H3K36me3	3
H3K79me2	4
hypomethylation	5
H3K9me3	6
H3K27me3	7
LADs	8
late-replicating	9

#### Computation time

**Fig 1:**
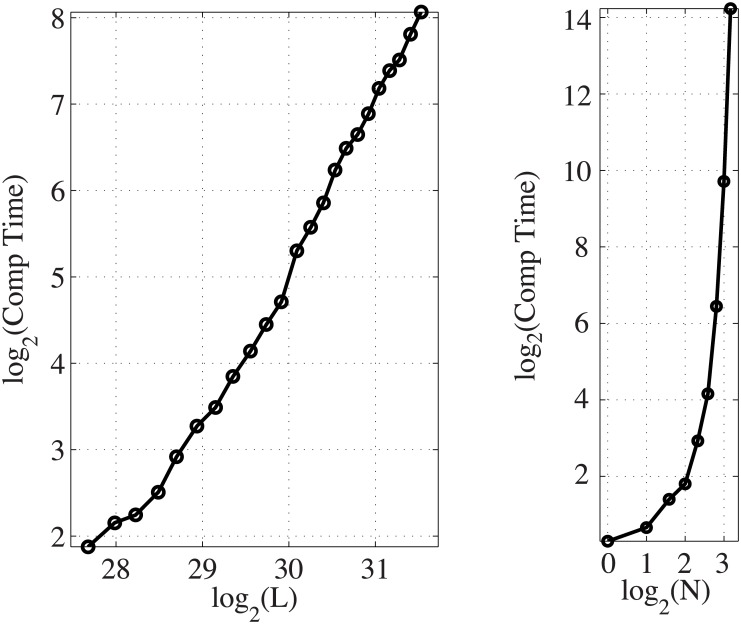
GINOM computation time experiments with simulated data. Left: Computation time v. *L* with fixed values of *N* = 5 and *n* = 2540. Right: Computation time v. *N* on chromosome 19 (*L* = 5.9 × 10^7^) with *n* = 2540.

First, we performed two computation time experiments running GINOM in MATLAB on a laptop with a 2.6 GHz processor. The computation times reported below for GINOM include the time necessary for data preprocessing as well as an unrestricted stepwise model selection. Fig 1 (left) shows how computation time scales with an increase in the length of the genome *L*, and Fig 1 (right) shows how computation time scales with an increase in the number of reference sets *N* considered in model selection. We also scaled the query sample size *n*, fixing everything else; however, we found that *n* only slightly affects the preprocessing stage and does not factor at all into model selection. Since the parts of GINOM that are influenced by *n* represent only a small fraction of the overall computation time, we omit the results of any computation time experiment versus *n*.

For the experiment in Fig 1 (left), we used *N* = 5 reference sets—specifically, sets 3, 4, 6, 7, and 8. For each value of *L*, we randomly generated ten query interval datasets, each of size *n* = 2540, with model terms *θ*_{6}_ = 1.0, *θ*_{7}_ = 0.5, *θ*_{6,7}_ = −1.0, and all other possible model terms set to equal zero. The parameter vector of this model is written simply as *θ* = (*θ*_{6}_, *θ*_{7}_, *θ*_{6,7}_)′ = (1.0, 0.5, − 1.0)′, dropping all of the zero-valued model terms. We used a sample size of *n* = 2540 because it is the same size as the real query interval dataset used later in the paper. We increased *L* systematically by starting with a domain G equal to all loci in chromosomes X and Y, and then appending an additional chromosome to G until the entire genome was considered. For each value of *L*, we ran GINOM with an unrestricted model selection for each of the ten replicates and averaged the computation time. From the plot in Fig 1 (left), we conclude that computation time scales on the order of *L*^2^. In this configuration, GINOM ran on the entire genome in an average of 268 seconds.

For the experiment in Fig 1 (right), we randomly generated one query interval dataset of size *n* = 2540 using the same value of *θ* as before with domain G equal to all loci in chromosome 19. We ran GINOM with unrestricted stepwise model selection nine times, each time considering an additional reference set until all nine reference sets in Table 1 were considered. From the plot in Fig 1 (right), we see that computation time increases rapidly with *N*. Therefore, for higher values of *N*, it becomes more beneficial to restrict the model selection in some way. For example, one could consider only lower-order terms like those with |*π*| ≤ 3, or one could restrict to a specific, predetermined list of biologically meaningful combinations. With the maximum value *N* = 9 in the experiment, GINOM ran on chromosome 19 in 5 hours and 21 minutes.

#### Performance of parameter estimation and model selection with convergence and identifiability study

**Fig 2:**
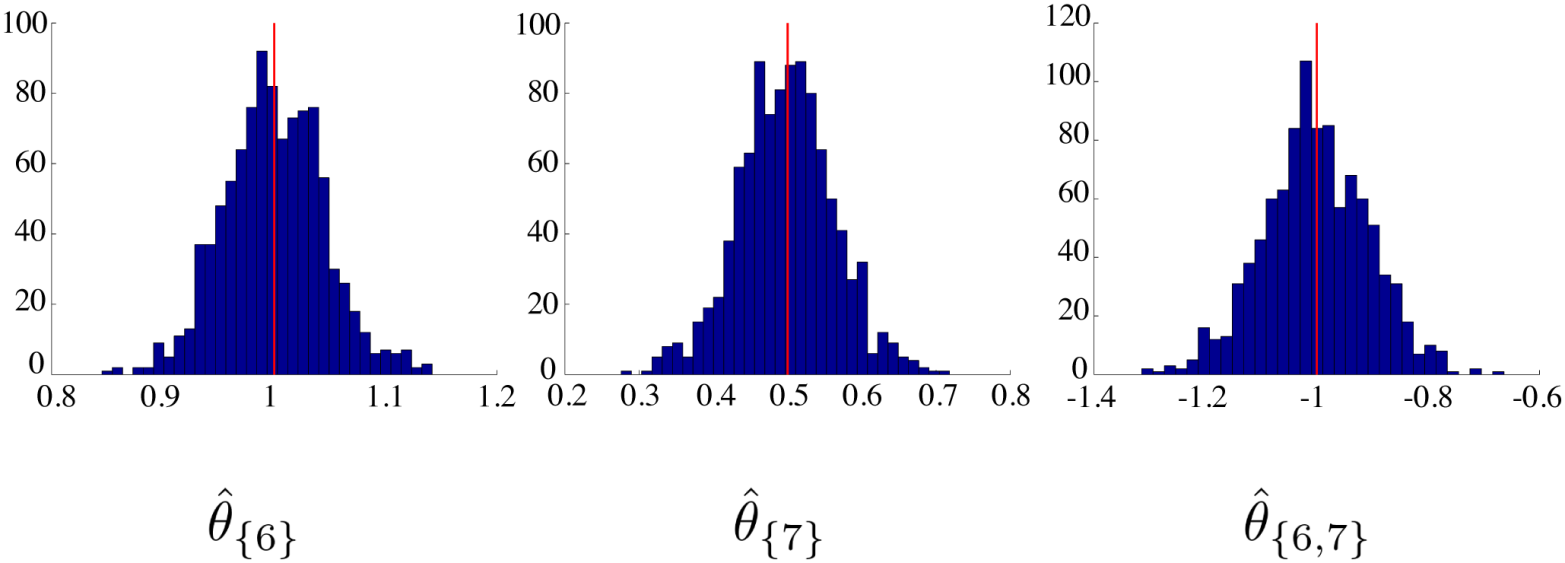
Histograms of estimated model parameter values. The components of $\hat{\theta }$ were computed from 1000 simulations of sample size *n* = 2540.

Next, we performed an experiment on chromosome 19 to compute an empirical distribution of $\hat{\theta }$ when fitting the true model to simulated data. This time, we randomly generated 1000 query interval datasets of fixed size *n* = 2540 using the same parameter vector *θ* = (*θ*_{6}_, *θ*_{7}_, *θ*_{6,7}_)′ = (1.0, 0.5, − 1.0)′. For each dataset, we fit the true model, i.e. we solved Eq (7) with all terms besides *θ*_{6}_, *θ*_{7}_, and *θ*_{6,7}_ constrained to be zero. Table 2 shows the mean and standard deviation of each component of $\hat{\theta }$, and Fig 2 shows the corresponding histogram for each component.

**Table 2:** GINOM parameter estimation performance on simulated data.

Model Term	True *θ*	Mean $\hat{\theta }$	Std. $\hat{\theta }$
{6}	1.0	0.9998	0.04486
{7}	0.5	0.4981	0.06459
{6, 7}	−1.0	−1.002	0.09500

The experiment was performed with sample size *n* = 2540 and 1000 replicates.

Using the same simulated data, we additionally ran the stepwise model selection for each of the 1000 datasets. In this experiment, we restricted the model selection to consider all possible combinations of reference sets 3, 4, 6, 7, and 8. Table 3 shows the top ten selected model configurations, percentage-wise. The true model configuration is shown in bold and is correctly selected 89.1% of the time. Each of the three correct model terms, *θ*_{6}_, *θ*_{7}_, and *θ*_{6,7}_, were selected 100% of the time, and no other incorrect model term was selected more than 1% of the time. In other words, the false negative rate for each correct model term was 0%, and the false positive rate for each incorrect model term was no more than 1% for this experiment. The rate of including at least one incorrect model term was 10.9%. The results of the simulation experiment as seen in Tables 2 and 3 provide not only a validation that the algorithms associated with GINOM are working properly but also a measure of error associated with estimation and model fitting in a scenario that incorporates the given reference sets.

**Table 3:** GINOM model selection performance for simulated data.

Selected Model	Perc. Selected	Req. Samp. Size
**{** **6** **}**, **{** **7** **}**, **{** **6**, **7** **}**	**89.1** **%**	**44**
{6}, {7}, {6, 7}, {4, 6, 8}	0.9%	1250
{6}, {7}, {6, 7}, {4, 7, 8}	0.7%	2300
{6}, {7}, {6, 7}, {7, 8}	0.7%	57
{6}, {7}, {6, 7}, {3, 4, 8}	0.6%	2000
{6}, {7}, {6, 7}, {3, 8}	0.6%	240
{6}, {7}, {6, 7}, {4, 8}	0.6%	900
{6}, {7}, {6, 7}, {6, 8}	0.6%	57
{6}, {7}, {6, 7}, {3, 4, 7}	0.5%	680
{6}, {7}, {6, 7}, {4, 7}	0.5%	500
Others	Remaining 5.2%	

The model selection was performed using the stepwise algorithm with BIC penalty on simulated data with sample size *n* = 2540 and 1000 replicates. The required sample size for 99% convergence was computed by varying *n* and computing the proportion of convergence over 5000 replicates for each value of *n*.

The estimation in Eq (7) fails to converge when at least one of the components of $\hat{\theta }$ goes to positive or negative infinity. Below, we list some of the conditions that must be met for convergence. The list is not exhaustive, as more complicated models can yield more complicated convergence criteria. The simplest condition for convergence is to have no component of *T* equal to 0, where *T* was introduced in Eq (5). That is, for each index set *π* included in the model, we need at least one query interval in the data set to simultaneously overlap all of the reference sets in *π*. Convergence also requires that, when there exist two model terms indexed by $\tilde{\pi }$ and *π* such that $\tilde{\pi }\subset \pi$, then *t*_*π*_ cannot equal $t_{\tilde{\pi }}$. As the sample size *n* increases, the probability of satisfying these criteria increases, and convergence is more certain.

The third column of Table 3 shows the minimum required sample size *n* to achieve a 99% convergence rate for that particular model configuration. To compute the convergence rate for a given *n* and a given model configuration, we first simulated 5000 query interval datasets of size *n* using the true model parameter values. Then, we computed the proportion of times that all components of $\hat{\theta }$ had an absolute value less than 5, i.e. the estimate did not drift off to positive or negative infinity. Fig B in (S1 Text) shows a plot of convergence percentage versus *n* for the first two model configurations listed in Table 3.

In addition to convergence, model identifiability is another technical consideration when analyzing the results of GINOM. When a model is not identifiable, the MLE is not unique. Unlike convergence, identifiability is not affected by the query sample size *n* but rather the locations of the reference sets in relation to one another. The condition necessary for identifiability is easiest to describe when considering a full model with *N* reference sets and *y* = 1. For example, the model that we used in all of our simulation experiments, where *θ* = (*θ*_{6}_, *θ*_{7}_, *θ*_{6,7}_)′, is a full model. This is because, for *N* = 2, the model terms consist of exactly all of the possible 2^*N*^ − 1 combinations of reference sets 6 and 7. For a general *N*, notice that the reference sets partition $G_{0}\left(1\right)$, the mappable part of the genome, into 2^*N*^ disjoint sets. If any of these disjoint sets are empty, i.e. they do not exist, then the full model is not identifiable. Continuing with our example, in order for this full model to be identifiable, the sets *R*_6_\*R*_7_, *R*_7_\*R*_6_, *R*_6_ ∩ *R*_7_, and $G_{0}\left(1\right)\backslash \left(R_{6}\cup R_{7}\right)$ must all be non-empty. In other words, *R*_6_ cannot be contained entirely within *R*_7_ (and vice versa), *R*_6_ and *R*_7_ cannot be disjoint, and the union of *R*_6_ and *R*_7_ cannot be $G_{0}\left(1\right)$. Indeed, this is the case for these reference sets. Furthermore, the full model with all *N* = 9 reference sets listed in Table 1 is identifiable in this sense when including all chromosomes in the domain, as all 2^9^ disjoint sets are non-empty. Identifiability becomes more complicated to describe when considering submodels and cases when *y* > 1, but it is easy to verify numerically in the data preprocessing stage of GINOM.

#### Comparison with current methods

We compared the analysis from GINOM with that of four other methods—BITS, MULTOVL, GAT, and GenometricCorr—on one simulated dataset. As before, we simulated a dataset of *n* = 2540 query intervals from the density in Eq (3) with *θ* = (*θ*_{6}_, *θ*_{7}_, *θ*_{6,7}_)′ = (1.0, 0.5, − 1.0)′. Since one of the methods, GAT, does not work for query intervals of length *y* = 1, we set each *y*_*i*_ equal to a randomly-selected query interval length from the real dataset used later in the paper, which does not contain any query intervals of length 1. We provide the results in (S1 Table).

Each of the four other methods performs their analysis in a pairwise fashion, where they compute query interval overlap statistics individually for each reference set. In order to compare GINOM directly to the other methods, we fit 9 models, each consisting of only one model term *π* = {*j*}, for *j* = 1, 2, …, 9. The differences in outputs between GINOM when run in a pairwise fashion and each of the four methods are due to different statistical tests as well as differences in data preprocessing techniques. On the other hand, these results were similar for all five methods in the sense that some significant effects were detected outside of the true effects of *R*_6_ and *R*_7_. For example, *R*_3_ was reported as a significant effect for all methods except GAT. This phenomenon is actually due to *R*_3_’s association with the enriched set *R*_6_ rather than an association with the query set itself. A large amount of *R*_3_ (45% of all *x* ∈ *R*_3_) is contained within *R*_6_, and thus, query interval enrichment due to the effect of *R*_6_ was additionally and falsely attributed to *R*_3_ in the pairwise analysis.

GINOM has a significant advantage over the other four methods in that it is not constrained to a pairwise analysis; rather, it can analyze the effects of all combinations of reference sets simultaneously. The stepwise model selection in GINOM is more likely to eliminate the false associations that arise from a pairwise analysis, outputting a simplified model configuration that best describes the data according to the BIC. When running GINOM a single time with an unrestricted stepwise model selection that considered all *N* = 9 reference sets, it recovered the true model with no false associations. The results of GINOM from running both the pairwise analysis as well as the stepwise model selection are shown in (S1 Table).

### A study of L1 insertion bias

We provide an application for GINOM using real data. For this example, we examine somatic insertions of the active Long INterspersed Element-1 (LINE-1 or L1) retrotransposon into the genome and test for insertion biases. LINEs are mobile elements present in many eukaryotes; their number can increase through a copy-and-paste mechanism and they have been implicated in several diseases [9]. Recently, somatic retrotransposition was identified as a frequent event in many human cancers and in the adult human brain [10–18]. A major biological question arising from this work is whether LINE-1, the active human retrotransposon, displays a bias in the locations of somatic retrotransposition. Various and sometimes contradictory biases for L1 retrotransposon insertions have been identified. Prior experimental work on the L1 protein machinery identified a retrotransposition bias for accessible DNA [19] and an L1 endonuclease recognition motif (TTAAAA) [20]. High-throughput studies of somatic retrotransposition uncovered a disproportionate bias towards affecting protein-coding genes in the brain and towards genes commonly mutated in cancer [11, 17]. Contradicting this view, high-throughput studies of germ-line retrotranspositions were identified to display depletion in protein-coding regions [21, 22]. One other interesting bias is that somatic L1 retrotranspositions in cancer are enriched towards regions that also display DNA hypomethylation [17]. We aim to rigorously test the biases associated with somatic L1 retrotranspositions. To do so, we compiled a dataset consisting of 2540 somatic L1 retrotranspositions available from a single tissue that were identified in lung cancer from two independent studies [13, 18]. The Helman *et al* study identified 363 somatic retrotransposition events in lung adenocarcinoma (39 events) and lung squamous cell carcinoma (324 events) from The Cancer Genome Atlas (TCGA) [13]. Tubio *et al* identified 2177 L1 retrotransposition events from various lung cancer sources including primary tumor samples, cell-lines, and TCGA samples [18].

Here, we examine the overlaps of the lung cancer somatic L1 retrotransposition query set: 2540 events (hg19 coordinates) with respect to nine curated reference sets that represent features of protein-coding genes, euchromatin, and heterochromatin. We reasoned that the ability of novel LINE-1 copies to insert themselves into a given region might be affected by this region’s accessibility; hence, we selected a set of genomic and epigenomic features that are known to be associated with genome accessibility and active transcription. The gene feature reference set includes RefSeq gene bodies (UCSC version update 10/06/15). Euchromatic tracks include broad peaks identified by the ENCODE project for histone marks H3K36me3, H3K79me2, and H3K4me3 in lung cancer cell-line A549 [23]. Our selected heterochromatic marks include additional ENCODE broad peaks for histone marks H3K9me3 and H3K27me3 in A549 cells [23], which have a role in repressing gene expression. We also included a track for two additional heterochromatic features, lamina associated domains (LADs) and late DNA replicating regions that, although defined in fibroblasts, are partially conserved between cell-types [24, 25]. Finally, we included a track for regions hypomethylated in cancer cells [26].

We compared our method with four other methods: BITS, MULTOVL, GAT, and GenometriCorr. However, unlike our method, which can examine multiple features simultaneously, these other methods test for significant overlap in a pairwise manner. Somatic L1 retrotranspositions in lung cancer displayed a bias for heterochromatic marks including H3K9me3, H3K27me3, lamina-associated domains (LADs), and late-replicating regions according to BITS, MULTOVL, GAT, and GenometricCorr by the Jaccard measure (S2 Table). In addition, consistent with Lee *et al.* [17], somatic L1 retrotransposition also displayed a preferential bias towards regions hypomethylated in cancer according to BITS, MULTOVL, GAT, and GenometricCorr by the Jaccard measure (S2 Table). Conversely, and consistent with studies of germ-line retrotransposition, somatic L1 insertions displayed a bias against gene regions and euchromatin marks including H3K4me3, H3K36me3, and H3K79me2 according to MULTOVL, GAT, and GenometricCorr by the Jaccard measure (S2 Table). Therefore, using the pairwise approach, we did not observe a bias of lung somatic L1 retrotranspositions towards gene regions. We hypothesized that our new method GINOM, which can examine combinations of overlaps, might be able to recover gene regions that account for previously observed biases of somatic retrotranspositions towards cancer mutations [17].

We examined the lung L1 somatic retrotransposition query set with respect to all nine reference sets using GINOM with the BIC penalty in model selection (see Model Selection subsection within Materials and methods section). Our null model is that of Eq (2) with $G_{0}\left(y\right)$ being the set of loci *x* such that *q*(*x*, *y*) would lie entirely within the mappable regions of G. GINOM reports significant model parameters and their associated MLE and *p*-value of the GLRT, and we show the results in Table 4.

**Table 4:** GINOM model selection on lung cancer L1 somatic retrotransposition data.

Model Term	Primary Effect	$\hat{\theta }$	*p*-value
{5}	Yes	0.7654	<10^−16^
{6}	Yes	0.3780	<10^−16^
{8}	Yes	0.4929	<10^−16^
{9}	Yes	0.4968	<10^−16^
{2, 7}	Yes	0.2528	1.675 × 10^−5^
{5, 8, 9}	No	−0.4497	1.432 × 10^−10^
{6, 7, 8}	No	−0.2907	5.480 × 10^−4^
{2, 4, 6, 9}	No	−1.668	0.001337
{2, 3, 4, 5, 6}	No	−1.985	0.003666

The experiment was performed on data consisting of lung cancer somatic L1 retrotranspositions as query intervals and the reference set collection: (1) H3K4me3, (2) genic regions, (3) H3K36me3, (4) H3K79me2, (5) hypomethylation, (6) H3K9me3, (7) H3K27me3, (8) LADs, (9) late-replicating.

For ease of interpretation, we label the significant model terms as either a *primary effect* or a *secondary effect*. A primary effect indexed by model term *π* is such that there exists no other significant model parameter indexed by $\tilde{\pi }$ where $\tilde{\pi }\subset \pi$. For example, model term {2, 7} is a primary effect because no model term with its index set contained in {2, 7} (e.g. either term {2} or term {7}) is significant (Table 4). A secondary effect is a model term that has at least one primary effect contained within its index set. For example, model term {6, 7, 8} is a secondary effect because primary effects {6} and {8} are contained within {6, 7, 8} (Table 4).

**Fig 3:**
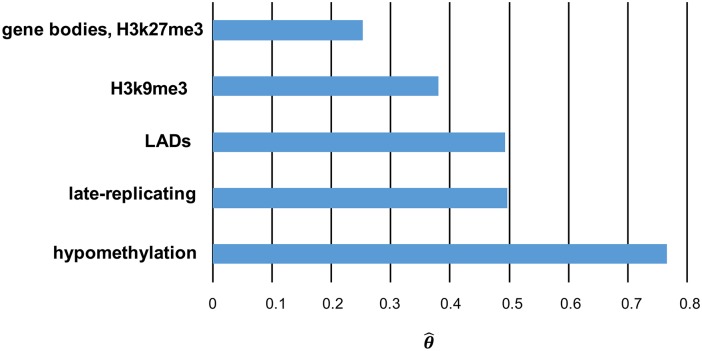
Primary effects of lung cancer somatic L1 retrotransposition. The results were output from GINOM model selection under BIC penalty.

The primary effects in the model selected by GINOM under the BIC penalty were hypomethylation, late-replicating, LADs, H3K9me3, and the combination of gene regions and H3K27me3 (Fig 3). Here, all primary effects showed enrichment with respect to the background, and, interestingly, the strongest of these enrichments occured in hypomethylation, model term {5}, which was consistent with Lee *et al.* [17] (Fig 3). With respect to $\tilde{x}$ in the background, the enrichment of a location *x* such that *q*(*x*, *y*) overlaps *R*_5_ only (and not any other reference set) is $h\left(x|y\right)={\hat{\theta }}_{\left\{5\right\}}=0.7654$. Therefore, the probability of a query interval *q*(*x*, *y*) is *e*^0.7654^ = 2.15 times that of $q(\tilde{x},y)$. The model was also able to recover gene regions when paired with the facultative heterochromatin mark H3K27me3, given by model term {2, 7}. The euchromatin marks were also frequently incorporated into secondary effects. For example, model term {2, 4, 6, 9}, which represents the combination of gene regions, H3K79me2, H3K9me3, and late-replicating regions, was assigned a parameter value of ${\hat{\theta }}_{\left\{2,4,6,9\right\}}=-1.668$. It therefore acts antagonistically to and with greater magnitude than the sum of the effects contained within it (terms {6} and {9}) so that the overall combined effect of *R*_2_, *R*_4_, *R*_6_, and *R*_9_ is negative. Specifically, for a location *x* such that *q*(*x*, *y*) simultaneously overlaps *R*_2_, *R*_4_, *R*_6_, and *R*_9_ only, the enrichment is given by $h\left(x|y\right)={\hat{\theta }}_{\left\{6\right\}}+{\hat{\theta }}_{\left\{9\right\}}+{\hat{\theta }}_{\left\{2,4,6,9\right\}}=-0.7932$. Therefore, such an *x* is depleted, with the probability of *q*(*x*, *y*) being *e*^−0.7932^ = 0.4524 times that of $q(\tilde{x},y)$ in the background, which is consistent with somatic transpositions being biased against euchromatin.

Finally, we sought to more closely examine the main effect resulting from the intersection of gene regions and H3K27me3. It was recently reported that L1 retrotranspositions display a bias towards cancer genes [17]. To test this in our dataset, we utilized the cancer gene lists from Lee *et al*, which include the COSMIC cancer gene census and the Memorial Sloan-Kettering Cancer Center cancer gene list (S3 Table). In the L1 lung cancer insertion dataset, we observed that 759 genes bodies overlap a somatic L1 retrotransposition. Of these, a significant proportion (37/759) are cancer genes (*p*-value = 0.027, hypergeometric test, S1 Text). We then looked more specifically at the L1 insertions in gene bodies marked by H3K27me3, which represented a significant main effect in the GINOM model. Of the 759 gene bodies that overlap a somatic L1 retrotransposition, 309 genes also intersect a H3K27me3 broad peak, and are significantly enriched for cancer genes (22/309, *p*-value 0.001, hypergeometric test, S1 Text). Hence, we speculate that L1 insertion bias towards cancer genes could be a consequence of L1 bias towards genes located within the facultative heterochromatin marked by H3K27me3.

Together our results reconcile some of the discrepancies in observations between somatic L1 retrotranspositions and germ-line retrotransposition events. Overall, somatic L1 retrotranspositions display a bias towards features typically associated with constitutive heterochromatin, similar to germline transposition events. However, some genes, e.g. cancer genes and genes marked by H3K27me3, also display a bias for somatic L1 transposition events in lung cancer.

### Conclusion

We developed a Genomic INterval Overlap Model that allows for the interrogation of significant associations between many genomic features simultaneously. Unlike prior methods, which test for associations in a pairwise manner, GINOM treats query interval location as a random variable of log-linear distribution with model terms formed from any possible combination, or interaction, of multiple reference sets. In this fashion, GINOM can uncover any higher-order interaction among reference sets that has a significant effect on query interval location. Through an implementation of a stepwise model selection routine, GINOM can handle an arbitrarily large number of reference sets simultaneously as input and subsequently output a reduced model that satisfies an optimal trade-off between number of model terms and likelihood value. The end result is a set of significant model terms with associated parameter values that defines a profile of query interval enrichment at a level of detail beyond that of pairwise comparisons.

To highlight this unique capability of GINOM, we fit the model using a query interval set of lung cancer somatic L1 retrotranspositions and a collection of reference sets representing protein-coding genes, euchromatin, and heterochromatin features. The output of GINOM indicates enrichment towards the individual heterochromatic reference sets, as do other current methods. However, GINOM uncovers more nuanced associations than the other methods by identifying a significant enrichment within genes only when paired with the H3K27me3 heterochromatic mark. Conversely, it also indicates depletion within genes when paired with certain euchromatic marks. The association of L1 somatic retrotranspositions and gene bodies, when marked by H3K27me3, is not recovered by other methods because they only consider pairwise associations.

Our results demonstrate GINOM’s ability to test for significance of interval overlap between multiple genomic features. As more data of this type becomes available, it will provide an effective method to screen for yet-uncharacterized higher-order associations between genomic features.

## Supporting information

S1 Table Analysis of results of competing methods on simulated data. We collect results from the following four methods: BITS, MULTOVL, GAT, and GenometricCorr. Additionally, we show results from GINOM when run on individual reference sets as well as when running stepwise model selection over all combinations of reference sets.
(XLSX)

S2 Table Analysis of results of competing methods on lung cancer somatic L1 retrotransposition data. We collect results from the following four methods: BITS, MULTOVL, GAT, and GenometricCorr.
(XLSX)

S3 Table Gene lists used in the cancer genes enrichment analysis. (XLSX)

S1 Text Additional details regarding hypothesis testing, model interpretation, maximum likelihood estimation, convergence, and cancer genes enrichment analysis (including R code). (PDF)
