# Supplementary material for: GINOM: A statistical framework for assessing interval overlap of multiple genomic features
Source: PLoS Comput Biol. 2017 Jun 15;13(6):e1005586. doi: 10.1371/journal.pcbi.1005586 (PMC5491313; doi:10.1371/journal.pcbi.1005586)
Supplement: S1 Text — (PDF) [file pcbi.1005586.s004.pdf]

Supplemental text to "GINOM: A Statistical Framework for Assessing Interval Overlap of Multiple Genomic Features"

Hypothesis Testing

A hypothesis test is designed to provide statistical evidence to select from a null and alternative hypothesis about the value of θ. Given a parameter space Θ and a null parameter space $\Theta_0 \subset \Theta$, the hypotheses are written as

$$H_0 : \theta \in \Theta_0$$
$$H_1 : \theta \in \Theta_0^C,$$

where $\Theta_0^C \subset \Theta$ represents the complement of the null parameter space within the larger space – the parameter space of the alternative hypothesis. Since our model is formulated from an exponential family of distributions, all the usual regularity conditions hold, and the application of the GLRT is standard as long as $\Theta_0 \subset \Theta$, i.e., as long as the parameter spaces are nested. In fact, all models and hypotheses we consider satisfy all regularity conditions needed for the MLE's and GLRT statistics to have their usual asymptotic distributions as the sample size n goes to infinity. The GLRT statistic is given by $D = -2\log(\Lambda)$, where Λ is defined as the following likelihood ratio:

$$\Lambda = \frac{\max_{\theta\in\Theta_0}\{f(\mathbf{x}|\mathbf{y},\theta)\}}{\max_{\theta\in\Theta}\{f(\mathbf{x}|\mathbf{y},\theta)\}},$$

where $\mathbf{x} = \{x_1, \ldots, x_n\}$ and $\mathbf{y} = \{y_1, \ldots, y_n\}$, and therefore,

$$D = 2\left(\max_{\theta\in\Theta}\{\ell(\theta)\} - \max_{\theta\in\Theta_0}\{\ell(\theta)\}\right).$$

To avoid confusion, we emphasize that the quantity D given here, although similar, is *not* the same as the deviance, which also usually bears the notation D but is typically considered within the context of general linear models (GLM's) instead. The formula for deviance can be obtained from the formula for D above by multiplying by -1 and replacing Θ_0 with Θ_s, the parameter space of a "saturated" model that would fit the data perfectly.

The GLRT statistic D given here is distributed asymptotically according to a $\chi^2(\nu)$ distribution, where the degrees of freedom $\nu = \dim(\Theta) - \dim(\Theta_0)$. A p-value can thus be obtained by comparing the GLRT statistic to the $\chi^2(\nu)$ distribution. For example, in order to test if f differs from f_0, i.e. to test the hypothesis given by

$$H_0 : \theta = \mathbf{0}$$
$$H_1 : \theta \neq \mathbf{0},$$

we let $\Theta_0 = \mathbf{0}$ and $\Theta = \mathbb{R}^d$, and, since $\ell(\mathbf{0}) = 0$, the GLRT statistic is given by

$$D = 2\hat{\theta}'T + 2\sum_{i=1}^{n} \log(c(\hat{\theta}, y_i)).$$

Here, $\nu = d - 0 = d$, and thus we obtain a p-value for the hypothesis test by comparing D to the $\chi^2(\nu)$ distribution.

We also use the GLRT to test whether each individual component of θ differs from zero or not. That is, for each of the d model parameters, we test the hypothesis

$$H_0 : \theta_\pi = 0$$
$$H_1 : \theta_\pi \neq 0$$

to see whether or not model term π has a significant effect. For this test, $\Theta_0 = \{\theta \in \mathbb{R}^d | \theta_\pi = 0\} = \mathbb{R}^{d-1}$, and $\Theta = \mathbb{R}^d$. In order to compute D with these parameter spaces, one must compute an MLE over each respective space. In other words, one must fit a size d model that includes all of the relevant model parameters and another size $d-1$ model that includes all relevant model parameters besides the π parameter. In this case $\nu = d - (d-1) = 1$, and thus we obtain the p-value for the π component of θ accordingly from the $\chi^2(1)$ distribution.

Model Interpretation

The values of the model parameters directly define a profile of query interval enrichment or depletion in regions of the genome that indicate overlap with one or multiple reference sets. Here we define an enrichment function from the model density function to aid in model interpretation. In the following, we drop the use of bold face in vectors. Define the column vectors

$$b(x|y)' = (r_{\{1\}}(x|y), r_{\{2\}}(x|y), \ldots, r_{\{N\}}(x|y))$$
$$\theta' = (\theta_\pi)$$
$$r(x|y)' = (r_\pi(x|y)) \text{ where } \pi \subset \{1, ..., N\}, \pi \neq \emptyset,$$

where the prime denotes transpose. The elements of the $(2^N - 1) \times 1$ vectors θ and $r(x|y)$ are indexed by the sets π, which can be in any arbitrary but fixed

ordering. Here, the parameters that are not included in the model are set equal to 0 in this full θ vector. The vector $b(x|y)$ is an element of $\mathcal{B} = \{0,1\}^N$, the set of all possible N-dimensional binary vectors. In terms of this notation, the model for a single query interval becomes

$$f(x|y) = c(\theta, y) f_0(x|y) \exp\{\theta' r(x|y)\}.$$

Since the elements of $r(x|y)$ are equal to either elements of $b(x|y)$ or products of such elements, we may regard $r(x|y)$ as a function of $b(x|y)$. We introduce ρ to denote the function $r(x|y) = \rho(b(x|y))$. Now the argument of the exponent in the model density above can be written as the function

$$g(x|y) = \theta' r(x|y) = \theta' \rho(b(x|y)).$$

Now, we define the enrichment function $h(x|y)$ as a ratio of how likely a query interval of length y is to have left endpoint x versus some other endpoint $\tilde{x}$, where the interval $q(\tilde{x}, y)$ would overlap none of the reference sets. Since $\rho(b(\tilde{x}|y))$ is equal to the zero vector, $g(\tilde{x}|y)$ is equal to 0, and thus the ratio $h(x|y) = f(x|y)/f(\tilde{x}|y)$ can be written as

$$h(x|y) = \frac{f_0(x|y)}{f_0(\tilde{x}|y)} e^{g(x|y)}.$$

If $h(x|y) > 1$ we say that the locus x is *enriched*, and if $0 < h(x|y) < 1$ we say that the locus x is *depleted*. Note that this function h is slightly different from the function h defined in the main text. The most common null distribution f_0 that is used in practical situations is the uniform distribution over a set $\mathcal{G}_0 \subseteq \mathcal{G}$. In this uniform case, assuming x and $\tilde{x}$ are both in $\mathcal{G}_0$, the fraction $f_0(x|y)/f_0(\tilde{x}|y)$ is equal to 1, and the enrichment function can be written as $h(x|y) = e^{g(x|y)}$. Now, equivalently, $g(x|y) > 0$ indicates enrichment and $g(x|y) < 0$ indicates depletion. For the remainder of this Section, we assume the common uniform case, using the function g as a means for model interpretation.

Since g can be considered a function of $b \in \mathcal{B}$, it takes only a finite number of values, which can be indexed by $\mathcal{B}$ in the following manner. For any $\beta \in \mathcal{B}$, the value of $g(x|y)$ over the set $\{x \in \mathcal{G} | b(x|y) = \beta\}$ is constant and denoted g_β, where $g_\beta = \theta' \rho(\beta)$. As an example, let us examine the values of g for the case when $N = 3$; in particular, we start with g_{100}. According to the definition above, g_{100} is the constant value that $g(x|y)$ takes over all x such that, for a given y, a query interval $q(x, y)$ would overlap reference set 1 but not reference sets 2 or 3. For $\beta = (1,0,0)'$, the only component of $\rho(\beta)$ that is non-zero is that of $r_{\{1\}}(x|y)$, and thus $g_{100} = \theta_{\{1\}}$. Now consider g_{110}. The non-zero components of $\rho(\beta)$ are given as $r_{\{1\}}(x|y)$, $r_{\{2\}}(x|y)$, and $r_{\{1,2\}}(x|y)$, and thus $g_{110} = \theta_{\{1\}} + \theta_{\{2\}} + \theta_{\{1,2\}}$. Continuing the example, g will take all of the following values:

$$g_{000} = 0, \quad g_{100} = \theta_{\{1\}}, \quad g_{010} = \theta_{\{2\}}, \quad g_{001} = \theta_{\{3\}},$$
$$g_{110} = \theta_{\{1\}} + \theta_{\{2\}} + \theta_{\{1,2\}},$$
$$g_{101} = \theta_{\{1\}} + \theta_{\{3\}} + \theta_{\{1,3\}},$$
$$g_{011} = \theta_{\{2\}} + \theta_{\{3\}} + \theta_{\{2,3\}},$$
$$g_{111} = \theta_{\{1\}} + \theta_{\{2\}} + \theta_{\{3\}} + \theta_{\{1,2\}} + \theta_{\{1,3\}} + \theta_{\{2,3\}} + \theta_{\{1,2,3\}}.$$

Now, we provide a simpler example where $N = 2$ with specific values of θ to help visualize and interpret a model. Let $\mathcal{G} = \{1, ..., 100\}$, $R_1 = \{21, \ldots, 60\}$, and $R_2 = \{41, \ldots, 80\}$; that is, we are given only two reference sets consisting of one interval each. Now, the number of possible model terms is $2^N - 1 = 3$ indexed by $\pi = \{1\}, \{2\}, \{1, 2\}$. For simplicity let us assume that $y = 1$ is the only admissible value of Y, and hence, we drop the use of conditional notation for all functions in this example. Furthermore, let $\theta = (\theta_{\{1\}}, \theta_{\{2\}}, \theta_{\{1,2\}})' = (0.5, 0.7, -0.2)'$, and let the null distribution f_0 be the discrete uniform distribution on $\mathcal{G}$. With this setup we have $f_0(x) = 0.01$ and $c(\theta) = 0.5966$, and thus the model equation is given by

$$\begin{aligned} f(x) &= f_0(x)c(\theta)\exp\{\theta' r(x)\} \\ &= 0.005966 \exp\{0.5 r_{\{1\}}(x) + 0.7 r_{\{2\}}(x) - 0.2 r_{\{1,2\}}(x)\}. \end{aligned}$$

For $N = 2$ and a query interval at x, the possible values of $\rho(b(x))$ are $\rho((0,0)') = (0,0,0)'$, $\rho((1,0)') = (1,0,0)'$, $\rho((0,1)') = (0,1,0)'$, and $\rho((1,1)') = (1,1,1)'$. Therefore, the values of the enrichment function $g(x) = \theta'\rho(b(x)) = \theta' r(x)$ are given by

$$\begin{gathered} g_{00} = 0, \quad g_{10} = \theta_{\{1\}} = 0.5, \quad g_{01} = \theta_{\{2\}} = 0.7, \\ g_{11} = \theta_{\{1\}} + \theta_{\{2\}} + \theta_{\{1,2\}} = 1.0. \end{gathered}$$

We can then interpret the above model as follows. Consider two particular locations x and $\tilde{x}$ where x lies in $R_1 \backslash R_2$ and $\tilde{x}$ lies in the background $\mathcal{G} \backslash (R_1 \cup R_2)$. A query interval is $e^{g_{10}} = e^{0.5} = 1.649$ times more likely to be located at x than at $\tilde{x}$. In other words, the effect of R_1, without contamination of the other reference set, shows enrichment at a level of 1.649 times that of the background. Similarly, we can say that the effect of R_2, without contamination of the other reference set, shows enrichment at a level of $e^{g_{01}} = e^{0.7} = 2.014$ times that of the background. Regarding the interaction term, since $\theta_{\{1,2\}} \neq 0$, the effect of overlapping both R_1 and R_2 does not equal the individual effects of R_1 and R_2 added together; rather, since $\theta_{\{1,2\}} = -0.2$, it is slightly lower than the additive effect of R_1 and R_2. In this case those loci that indicate an overlap with both R_1 and R_2 show enrichment at a level of $e^{g_{11}} = e^{1.0} = 2.718$ times that of the background.

Fig A shows a histogram of $n = 10000$ i.i.d. query interval starting locations simulated from the above distribution function $f(x)$. The histogram contains 100 bins, one for each locus $x \in \mathcal{G}$. Given n, the red dots represent the expected number of data points located in each bin under the model function f, and the green dots represent the expected number in each bin under the null distribution f_0. Beneath the histogram bars, the locations of R_1 and R_2 are shown in cyan and magenta, respectively. Table A shows the MLE over the full space $\mathbb{R}^3$ as well as the p-values associated with each component of θ. For this example, the p-values are all highly significant because (a) the data has been simulated directly from the model and (b) the sample size n is relatively high compared to the number of model parameters.

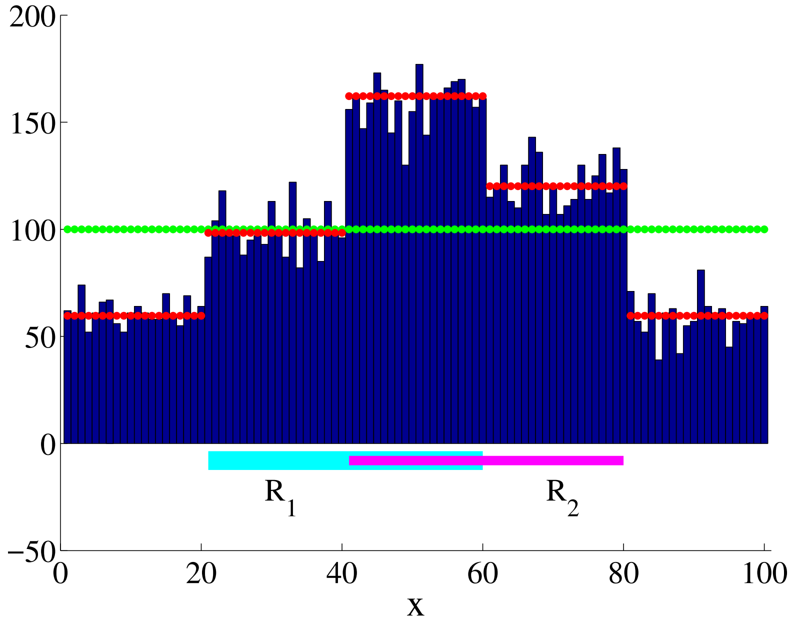

Figure A: **Example: histogram of simulated data.** Histogram of $\{x_i\}$, simulated data from model with $N = 2$, $y_i = 1$ for all i, and θ given by $\theta_{\{1\}} = 0.5$, $\theta_{\{2\}} = 0.7$, and $\theta_{\{1,2\}} = -0.2$. Reference sets R_1 and R_2 are shown and labeled. The expected number of counts per bin under the model function are shown in red, and the expected number of counts per bin under f_0 are shown in green.

Table A: **Example: Result of parameter estimation.**

Model Term	$\hat{\theta}$	p-value
$\{1\}$	0.4952	$< 10^{-16}$
$\{2\}$	0.7059	$< 10^{-16}$
$\{1,2\}$	-0.2268	1.189×10^{-8}

Computational Details of Maximum Likelihood Estimation

Here, we focus on how to write Eqn. 6 of the main text, its gradient, and its Hessian in terms of matrix multiplication in order to efficiently compute the optimization in Eqn. 7 as well as approximate confidence intervals for each component of θ. The main computational burden in Eqn. 6 is attributed to calculating the normalizing constant $c(\theta, y)$, and thus we address this issue first. Continuing with our notation from the previous Section, the normalizing constant may be

computed as follows.

$$\begin{aligned}\frac{1}{c(\theta, y)} &= \sum_{x \in \mathcal{G}} f_0(x|y) \exp\{\theta' r(x|y)\} \\ &= \sum_{b \in \mathcal{B}} \sum_{x: b(x|y)=b} f_0(x|y) \exp\{\theta' r(x|y)\} \\ &= \sum_{b \in \mathcal{B}} \sum_{x: b(x|y)=b} f_0(x|y) \exp\{\theta' \rho(b(x|y))\} \\ &= \sum_{b \in \mathcal{B}} \exp\{\theta' \rho(b)\} \sum_{x: b(x|y)=b} f_0(x|y) \\ &= \sum_{b \in \mathcal{B}} \exp\{\theta' \rho(b)\} S_{yb},\end{aligned}$$

where we define

$$S_{yb} = \sum_{x: b(x|y)=b} f_0(x|y).$$

The last summation in the sequence above may be expressed in terms of matrix products as follows.

$$\frac{1}{c(\theta)} \equiv \left(\frac{1}{c(\theta, y)} \right)_{y \in \mathcal{Y}} = S \exp\{Q\theta\},$$

where S is defined above and Q is the matrix with entries $Q_{b\pi} = \rho(b)_\pi$, where $\rho(b)_\pi$ is the π coordinate of $\rho(b)$. The set $\mathcal{Y}$ is the set of y values for which we desire to compute $c(\theta, y)$. For the remainder of the analysis, we let $\mathcal{Y}$ be the set of d distinct values found in the data $y_1, y_2, \ldots, y_n$. The components of the $d \times 1$ vector $c(\theta)$ consist of the values $c(\theta, y)$, $y \in \mathcal{Y}$.

Here, we have written the normalizing constant in such a way so that, once we have created the matrices S and Q, we can compute all the desired values of $c(\theta, y)$ in one line of code. The optimization in Eqn. 7 then simplifies computationally because we only have to compute S and Q once upfront and use them as input to any numerical function optimizer. Likewise, during any model selection procedure, the same matrices S and Q suffice for all the models we might consider; it is simply a matter of setting the components of θ to zero for those terms not included in the model.

Now the question remains of how we create the matrices Q and S. The $(2^N) \times (2^N - 1)$ matrix Q does not depend on the data and is easily created with the details depending on the particular ordering chosen for the subsets π. In lieu of providing an algorithm for computing Q, in Table B we show an example of its value when $N = 3$. Recall that the rows are indexed by $b \in \mathcal{B}^N$ and the columns are indexed by $\pi \subset \{1, 2, ..., N\}$.

To create the $d \times 2^N$ matrix S, we initialize all the elements to be zero, and then make one pass through all the possible pairs (x, y), $x \in \mathcal{G}$, $y \in \mathcal{Y}$, for each pair (x, y) incrementing the appropriate element of S as follows:

$$S(y, b(x|y)) = S(y, b(x|y)) + f_0(x|y),$$

Table B: **The value of the matrix Q when $N = 3$.**

	$\{1\}$	$\{2\}$	$\{3\}$	$\{1,2\}$	$\{1,3\}$	$\{2,3\}$	$\{1,2,3\}$
000	0	0	0	0	0	0	0
100	1	0	0	0	0	0	0
010	0	1	0	0	0	0	0
001	0	0	1	0	0	0	0
110	1	1	0	1	0	0	0
101	1	0	1	0	1	0	0
011	0	1	1	0	0	1	0
111	1	1	1	1	1	1	1

where for convenience we have used functional notation instead of subscripts, i.e. $S(y, b) \equiv S_{yb}$. When either $\mathcal{G}$ or $\mathcal{Y}$ is a large set, computing S in this fashion can be slow. In the appendix we provide a fast algorithm to compute S in the special case when f_0 is the discrete uniform distribution defined over a non-empty subset $\mathcal{M} \subseteq \mathcal{G}$. For example $\mathcal{M}$ could be all loci of the genome that have non-zero mappability.

The remaining quantity to compute in $\ell(\theta)$ is the vector T. In order to compute T, one first computes $b(x_i|y_i)$ for each query interval in the data. The vector T' is now given by the column sum of the $n \times (2^N - 1)$ matrix that has row i equal to $Q(b(x_i|y_i), \cdot)$, where we again for convenience have used functional notation instead of subscripts.

We can now write down $\ell(\theta)$, its gradient, and its Hessian in terms of matrix multiplication for ease of computation, but first we modify the notation for S to avoid confusion. The entries of S were denoted S_{yb} where y denoted one of the distinct values of y in the data. This notation becomes confusing when trying to distinguish between y_i, the i-th data value, and the i-th among the *distinct* data values. So instead we let the distinct data values be denoted $\tilde{y}_k$, $k = 1, 2, \ldots, d$, and index the entries of S as S_{kb}, which is the value (formerly denoted) $S_{\tilde{y}_k b}$ corresponding to the k-th distinct data value. Let m_k be the multiplicity of $\tilde{y}_k$, the number of times the value $\tilde{y}_k$ occurs in the data.

In this modified notation, the formula for $c(\theta, y)$ becomes

$$\frac{1}{c(\theta, \tilde{y}_k)} = \sum_b S_{kb} \exp\left(\textstyle\sum_\pi Q_{b\pi}\theta_\pi\right),$$

which leads to the same matrix expression as obtained earlier:

$$\left(\frac{1}{c(\theta, \tilde{y}_k)}\right)_{1 \le k \le d} = S \exp(Q\theta).$$

The log-likelihood function $\ell(\theta)$ is

$$\begin{aligned}\ell(\theta) &= \sum_{k=1}^{d} m_k \log c(\theta, \tilde{y}_k) + \theta' T \\ &= \theta' T - \sum_k m_k \log \left\{ \sum_b S_{kb} \exp \left(\sum_\pi Q_{b\pi} \theta_\pi \right) \right\} \\ &= \theta' T - m \log(S \exp(Q\theta)),\end{aligned}$$

where $m = (m_k)$ is a row vector.

From the above equation, the gradient can now be obtained as

$$\begin{aligned}\frac{\partial \ell(\theta)}{\partial \theta_\xi} &= T_\xi - \sum_k m_k \left(\frac{\sum_b Q_{b\xi} S_{kb} \exp\left(\sum_\pi Q_{b\pi}\theta_\pi\right)}{\sum_b S_{kb} \exp\left(\sum_\pi Q_{b\pi}\theta_\pi\right)} \right) \\ &= T_\xi - \sum_{k,b} m_k P_{kb} Q_{b\xi},\end{aligned}$$

which in matrix notation becomes

$$\left(\frac{\partial \ell(\theta)}{\partial \theta} \right)' = (\nabla \ell(\theta))' = T' - mPQ\,.$$

Here we have introduced the matrix $P = P(\theta)$ with entries

$$P_{kb} = \frac{S_{kb} \exp\left(\sum_\pi Q_{b\pi}\theta_\pi\right)}{\sum_b S_{kb} \exp\left(\sum_\pi Q_{b\pi}\theta_\pi\right)} = c(\theta, \tilde{y}_k) S_{kb} \exp\left(\sum_\pi Q_{b\pi}\theta_\pi \right),$$

which in matrix notation becomes

$$P = \mathrm{diag}(c(\theta)) S \mathrm{diag}(\exp(Q\theta)).$$

Note that the rows of P are probability vectors; they are nonnegative and sum to one, and in particular, $P_{kb} = P\{b(X|Y) = b \,|\, Y = \tilde{y}_k\}$. By explicitly defining the formula for gradient computation, a built-in function optimizer can usually make use of it to substantially speed up the optimization.

The Fisher information matrix is given by the negative of the Hessian matrix, and after some calculation, it can be obtained as

$$\begin{aligned}-\frac{\partial^2 \ell(\theta)}{\partial \theta_\xi \partial \theta_\lambda} &= \sum_\ell m_\ell \left[\sum_b P_{\ell b} Q_{b\xi} Q_{b\lambda} \right. \\ &\quad \left. - \left(\sum_b P_{\ell b} Q_{b\xi} \right) \left(\sum_b P_{\ell b} Q_{b\lambda} \right) \right] \\ &= \sum_b Q_{b\xi} \left(\sum_\ell m_\ell P_{\ell b} \right) Q_{b\lambda} \\ &\quad - \sum_\ell m_\ell (PQ)_{\ell\xi} (PQ)_{\ell\lambda} \\ &= \left(Q' \mathrm{diag}(mP) Q - (PQ)' \mathrm{diag}(m)(PQ) \right)_{\xi\lambda},\end{aligned}$$

where, for any row or column vector v, the matrix diag(v) denotes the diagonal matrix with the components of v along the diagonal. The observed Fisher information matrix $\hat{I}$ is used to obtain approximate confidence intervals for each component of θ and is given by the Fisher information matrix evaluated at $\theta = \hat{\theta}$. Since all regularity conditions apply, $\hat{\theta} \sim \text{ApproxNormal}(\theta, \hat{I}^{-1})$, and an approximate confidence interval for the model parameter θ_π at the level defined by the z-score z^* is given by

$$\left(\hat{\theta}_\pi - z^* \sqrt{(\hat{I}^{-1})_{\pi\pi}}, \hat{\theta}_\pi + z^* \sqrt{(\hat{I}^{-1})_{\pi\pi}}\right),$$

where $(\hat{I}^{-1})_{\pi\pi}$ is the diagonal entry of $\hat{I}^{-1}$ corresponding to the π'th model term.

Note: we use MATLAB for the implementation of GINOM; in particular we use the built-in function `fminunc` for unconstrained optimization and the built-in function `fmincon` for constrained optimization when necessary.

Convergence Plots

In Fig. B we show two plots of percent convergence versus the query sample size n for a given model configuration. Each data point in the plots below was generated by computing the proportion of times the estimation converged using 5000 simulated datasets of size n. Each dataset was simulated from the true model given in the main text using terms $\{6\}, \{7\}, \{6, 7\}$. The top plot shows the convergence behavior when fitting the true model, and the bottom plot shows the convergence behavior when fitting the model with terms $\{6\}, \{7\}, \{6, 7\}, \{4, 6, 8\}$ – the first incorrect model given in line two of Table 3 in the main text.

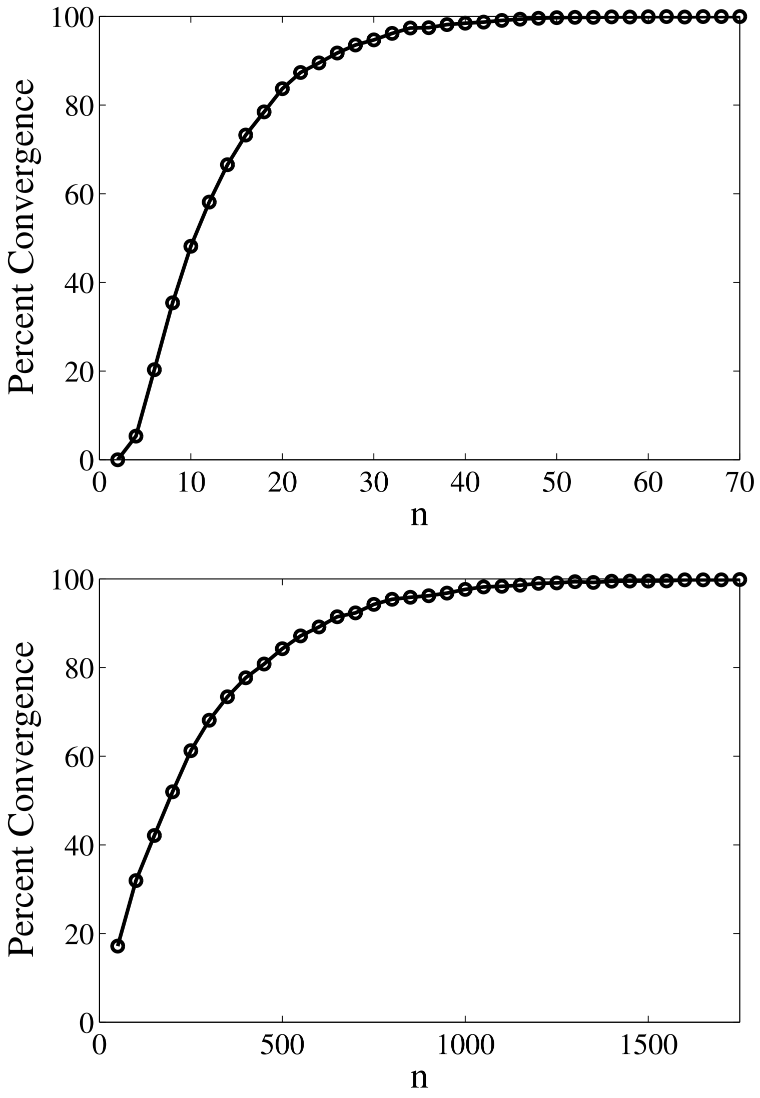

Figure B: **Percent convergence versus sample size** n. The top panel shows the behavior when fitting the true model, and the bottom panel shows the behavior when fitting an incorrect model.
